# Stress Influences the Effect of Obsessive-Compulsive Symptoms on Emotion Regulation

**DOI:** 10.3389/fpsyt.2020.594541

**Published:** 2021-01-20

**Authors:** Sónia Ferreira, Beatriz Couto, Mafalda Sousa, Rita Vieira, Nuno Sousa, Maria Picó-Pérez, Pedro Morgado

**Affiliations:** ^1^Life and Health Sciences Research Institute (ICVS), School of Medicine, University of Minho, Braga, Portugal; ^2^ICVS-3Bs PT Government Associate Laboratory, Braga, Portugal; ^3^Clinical Academic Center-Braga (2CA), Braga, Portugal

**Keywords:** OCD, obsessive-compulsive disorder, PSS-10, ERQ, OCI-R, cognitive

## Abstract

Obsessive-compulsive disorder (OCD) is associated with emotion regulation impairments, namely the frequent use of maladaptive strategies such as suppression and the decreased use of reappraisal strategies. Additionally, these patients exhibit elevated stress levels. Since stress exposure affects emotion regulation abilities, stress might influence the relationship between obsessive-compulsive symptoms and emotion regulation. In this study, we explored the effects of stress and obsessive-compulsive symptoms on emotion regulation in a sample of healthy and OCD individuals. We used self-reported psychometric scales to measure stress levels, obsessive-compulsive symptoms, and emotion reappraisal and suppression skills. We applied multiple regression and mediation analyses. Our results demonstrated that increased reappraisal scores were associated with higher suppression scores. Additionally, elevated stress values predicted increased scores for suppression and decreased scores for reappraisal. Furthermore, the reappraisal abilities resulted from a combination of a direct effect of obsessive-compulsive symptoms and an indirect effect of obsessive-compulsive symptoms mediated by stress. The reliance on suppression strategies and the difficulty in using reappraisal approaches are explained by stress levels and are not directly explained by obsessive-compulsive symptoms. This study highlights the necessity of targeting stress in current therapy-based treatments for OCD.

## Introduction

Obsessive-compulsive disorder (OCD) may arise from an interplay between genetic and environmental risk factors, namely exposure to stressful and traumatic life events (1, 2). Moreover, increases in general stress (e.g., job loss and family disease) and changes in routines throughout life are features associated with the development (3) and severity (4, 5) of OCD. OCD is characterized by elevated levels of anxiety and distress elicited by the presence of intrusive thoughts (obsessions) (6). The enhanced levels of distress might increase the hypothalamic-pituitary-adrenal (HPA) axis function resulting in an augmented stress response (7–9). In line with this assumption, previous studies have found correlations between perceived stress levels and obsessive-compulsive symptoms in OCD and healthy individuals (10–12), and between cortisol responses and obsessive-compulsive symptoms in healthy individuals (13). Additionally, other researchers demonstrated that increased cortisol levels are a hallmark of OCD also suggesting the hyperactivity of the HPA axis (10, 14), although contradictory findings were also found (11). Furthermore, brain anatomical and functional alterations in the striatum (caudate and putamen), hippocampus, amygdala, and medial and orbitofrontal prefrontal cortices have been reported for OCD and stress, suggesting that stress may exacerbate the bias toward habitual and ritualistic compulsive behaviors in OCD patients (1, 15–18).

OCD is also linked to emotional regulation deficits (19–21). Past research has shown that OCD patients frequently suppress their emotions instead of using more beneficial reappraisal strategies (22–26). The constant use of suppression has counterproductive effects leading to more distress and intrusive thoughts (27, 28). Cognitive reappraisal consists of the alteration of the initial experiencing of affective stimuli. Two main strategies are commonly used for cognitive reappraisal: (1) reinterpretation—interpretation of the stimulus to achieve a more positive/pleasant connotation; (2) distancing—visualizing the stimulus from the perspective of an unrelated observer or an unreal situation. For example, OCD patients can reinterpret their intrusive thoughts as a common event that occurs in everyone's life. Additionally, OCD patients can distance themselves from the consequences of their intrusive thoughts by thinking of them as not real. Expressive suppression relies on the inhibition of behavioral and emotional responses in the presence of affective stimuli (29–32). The emotional appraisal and regulation processes are linked to stress mechanisms. Acute and social stressors lead to the engagement of maladaptive emotion regulation strategies such as worry and rumination (33). Thus, the chronic use of these strategies might in turn augment the stress response. Indeed, maladaptive emotion regulation strategies have been associated with increased stress responses (33, 34), while reappraisal leads to enhanced stress recovery (34) in healthy participants and individuals with anxiety disorders. A recent meta-analysis also reported that reappraisal of fear/negative emotions induced by stressful tasks decreases the heart rate in healthy individuals (35). Moreover, emotion regulation difficulties translate into decreased heart rate variability (36, 37), a well-known biomarker of stress (38). Lastly, diminished cortisol and perceived stress levels in response to an acute stressor were observed after cognitive-behavioral stress management (39). These authors reported that the alterations in stress response were associated with changes in emotion appraisal.

In this way, stress may play a significant role in the relationship between OCD and emotion regulation. In this study, we aim to investigate the impact of stress and obsessive-compulsive (OC) symptoms on emotion regulation in a sample of non-psychiatric and OCD individuals using psychometric instruments. Based on the previous literature, we assume that higher scores for stress and OC symptoms are associated with less effective use of emotion reappraisal and enhanced use of emotion suppression strategies. Furthermore, past evidence suggests a link between stress and OC symptoms. Thus, we hypothesize that stress mediates the effect of OC symptoms on emotion regulation. This study elucidates the role of stress on OCD providing new recommendations for current psychotherapy approaches.

## Materials and Methods

### Participants

We included OCD patients and non-psychiatric control participants in this study. These groups of participants with low and high OC symptoms were recruited to have a wider range of OC symptomatology. OCD patients were recruited at the Psychiatry Unit of *Hospital de Braga* (Braga, Portugal) and diagnosed by a psychiatrist (PM) according to the Diagnostic and Statistical Manual of Mental Disorders (DSM-5) criteria. The patients were under treatment as usual or were treatment naïve. We excluded patients with comorbid psychiatric disorders or a history of neurological disorders. The control participants were recruited among the local community according to the age, gender, and education level of the patients, did not have a history of psychiatric/neurological disorders, and were not taking psychiatric medication.

All participants signed an informed consent. The study was approved by the ethics committees of *Hospital de Braga* (*Comissão de Ética para a Saúde*), Braga, Portugal, and University of Minho (*Subcomissão de Ética para as Ciências da Vida e da Saúde*), Braga, Portugal, and respected the Declaration of Helsinki principles.

### Psychometric Evaluation

The Yale-Brown Obsessive Compulsive Scale (Y-BOCS) was applied to evaluate the disease severity in OCD patients (40–42). We also applied the Emotion Regulation Questionnaire (ERQ) to measure reappraisal and suppression abilities (43, 44). The Obsessive-Compulsive Inventory-Revised (OCI-R) was also used to measure OCD severity and dimensions (washing, checking, ordering, hoarding, obsessing, and neutralizing subscales) (45, 46). The 10-items Perceived Stress Scale (PSS-10) was also applied to quantify self-perceived stress levels (10, 47, 48). PSS-10 measures prolonged psychological stress experienced in the month preceding the scale application.

### Statistical Analysis

The statistical analysis was performed with JASP (version 0.11.1; JASP Team, University of Amsterdam, The Netherlands). *P*-values under 0.05 were considered statistically significant. All statistical tests were two-tailed. The assumption of normality was assessed with the Shapiro-Wilk test. The Cohen's *d* effect size was calculated for all results: 0.2 ≤ *d* < 0.5 small effect; 0.5 ≤ *d* < 0.8 medium effect; *d* ≥ 0.8 large effect (49).

First, we evaluated differences in demographic (age, gender, and education) and psychometric (ERQ reappraisal and suppression, PSS-10, and OCI-R total and subscales) variables between the OCD and control group using independent samples *t*-tests for parametric variables and the Mann-Whitney [*U*] test for non-parametric variables (the chi-squared test was used for the categorical variable gender [χ^2^]). We used Bonferroni correction for multiple comparisons in the OCI-R subscales (washing, checking, ordering, hoarding, obsessing, and neutralizing).

Moreover, we explored the association among the variables (age, education, ERQ reappraisal and suppression, PSS-10, and OCI-R total) for all participants and within each group (OCD and control) using Pearson's or Spearman's correlation, depending on the normality of the variables. We used Bonferroni correction to correct for multiple comparisons.

After, we used two multiple regression models to study which demographic and psychometric variables predicted the ERQ reappraisal and ERQ suppression scores in the total sample. We tested the following predictors: age, gender, education, PSS-10, OCI-R total, and ERQ reappraisal/ERQ suppression. The assumptions of normality, linearity, and homoscedasticity were verified by visual inspection of the Q-Q and residuals-predicted plots. Correlations between residuals and multicollinearity were verified with the Durbin-Watson value (between 1.5 and 2.5) and tolerance (>0.1) and variance inflation factor (<10) values (50, 51).

Lastly, we performed a mediation analysis to understand if the OCI-R total score (predictor variable) predicted the ERQ reappraisal and suppression scores (outcome variables) when mediated by the PSS-10 score (mediator variable), using age, gender, and education as background confounders. We followed the assumptions for mediation analyses defined by Kenny and colleagues (52–54): the predictor variable influences the mediator variable; the mediator variable affects the outcome variable when controlling for the predictor variable. The influence of the predictor variable on the outcome variable is no longer a requirement for mediation analysis according to these authors. We applied the bias-corrected percentile bootstrap method with 1,000 replications. The use of bootstrapping in mediation analysis consists of a non-parametric method to estimate the sampling distribution of indirect effects without prior assumptions of the distribution shape, providing higher statistical power and more accurate estimation of confidence intervals than standard methods (55–57). This analysis was performed using the total sample. We assessed direct, indirect, and total effects of the OCI-R score on the ERQ reappraisal/suppression score. The indirect effect represents the amount of mediation by the PSS-10 score and the total effect result from the sum of direct and indirect effects (57, 58).

## Results

We included 43 OCD patients and 22 control participants. One OCD patient was excluded because he/she did not fill the OCI-R scale. Three patients were treatment naïve, 3 patients were not under medication, and the other patients were taking psychotropic medication (clomipramine, fluoxetine, fluvoxamine, sertraline, or escitalopram). Nine patients were being treated with psychotherapy (13 patients with missing information).

Table 1 contains the descriptive and statistical values for demographic and psychometric data. OCD and control groups were not different in terms of age, gender ratio, and education level. Additionally, we observed statistically significant increases in the PSS-10 score, and the OCI-R total, washing, checking, and obsessing scores in the OCD group. Moreover, OCD participants had decreased scores for the ERQ reappraisal subscale.

**Table 1:** Description of the demographic and psychometric variables for the obsessive-compulsive and control group and for the whole sample, and representation of the statistical differences between groups (independent samples *t*-test, Mann-Whitney test [*U*], and chi-squared test [*X*^2^]; *p*_bonf_ - *p*-value after Bonferroni correction; *d*—Cohen's effect size).

	**OCD** ** (*n* = 42)**	**Control** ** (*n* = 22)**	**Total sample** ** (*n* = 64)**	**Statistical results between groups (OCD vs. control)**
Age (years)	27.0 (13.7)	24.0 (15.2)	27.0 (15.2)	*U* = 392.00; *p* = 0.325; *d* = 0.25
Gender (F | M)	27|15	13|9	40|24	$X_{\left(1\right)}^{2}$ = 0.17; *p* = 0.683; *d* = 0.10
Education (years)	13.5 (4.7)	13.0 (3.7)	13.5 (4.2)	*U* = 431.50; *p* = 0.668; *d* = 0.11
Y-BOCS^†^				
Total	28.0 (5.0)	–	–	–
Obsessions	13.0 (5.0)	–	–	–
Compulsions	14.0 (3.0)	–	–	–
ERQ				
Reappraisal	24.9 ± 9.5	30.1 ± 7.9	29.0 (12.5)	*t*_(62)_ = −2.19; *p* = 0.032; *d* = −0.58^*^
Suppression	14.3 ± 5.1	15.0 ± 5.9	14.5 ± 5.4	*t*_(62)_ = −0.53; *p* = 0.595; *d* = −0.14
PSS-10	22.3 ± 8.0	15.4 ± 7.2	19.9 ± 8.3	*t*_(62)_ = 3.40; *p* = 0.001; *d* = 0.89^*^
OCI-R				
Total	31.8 ± 14.0	15.4 ± 10.3	26.2 ± 15.0	*t*_(62)_ = 4.82; *p* = 9.777 × 10^-6^; *d* = 1.27^*^
Washing	4.5 (6.0)	1.0 (1.7)	2.0 (5.2)	*U* = 175.50; *p*_bonf_ = 2.654 × 10^-4^; *d* = 1.17^*^
Checking	4.0 (6.0)	1.5 (2.0)	3.0 (4.5)	*U* = 190.00; *p*_bonf_ = 7.140 × 10^-4^; *d* = 1.10^*^
Ordering	5.0 (5.0)	4.0 (4.0)	4.0 (5.2)	*U* = 349.00; *p*_bonf_ = 0.660; *d* = 0.41
Hoarding	3.0 (4.0)	3.0 (3.5)	3.0 (4.0)	*U* = 464.50; *p*_bonf_ = 1.000; *d* = 0.01
Obsessing	8.0 (6.0)	2.5 (4.5)	5.5 (7.0)	*U* = 135.00; *p*_bonf_ = 2.128 × 10^-5^; *d* = 1.42^*^
Neutralizing	3.0 (7.0)	1.0 (2.0)	2.0 (6.0)	*U* = 292.00; *p*_bonf_ = 0.090; *d* = 0.63

Data represents mean ± standard deviation for normally distributed variables and median (interquartile range) for the other variables; OCD, obsessive-compulsive disorder; F, female; M, male; Y-BOCS, Yale-Brown Obsessive Compulsive Scale; ERQ, Emotion Regulation Questionnaire; PSS-10, Perceived Stress Scale (10 items); OCI-R, Obsessive-Compulsive Inventory-Revised;

† Four patients with missing data;

* *Statistically significant differences between groups*.

Table 2 summarizes Pearson's and Spearman's correlation results for the complete sample. We observed a positive correlation between the OCI-R and PSS-10 scores. Within the OCD group, we found a positive correlation between the OCI-R and PSS-10 scores (Supplementary Table 1). For the control group, we did not detect significant correlations after correcting for multiple comparisons (Supplementary Table 2). However, the correlation between the OCI-R and PSS-10 scores was also statistically significant in the control groups with the uncorrected *p*-value (Supplementary Table 2).

**Table 2:** Results of Pearson's (*rp*; normally distributed variables) and Spearman's (*rs*; variables not normally distributed) correlation among demographic and psychometric variables for the complete sample (*p*_bonf_ - *p*-value after Bonferroni correction; *d*—Cohen's effect size).

	**Education (years)**	**ERQ reappraisal**	**ERQ suppression**	**PSS-10**	**OCI-R total**
Age (years)	*rs* = −0.14, *p*_bonf_ = 1.000 *p* = 0.254; *d* = −0.28	*rs* = 6.31 × 10^−4^, *p*_bonf_ = 1.000 *p* = 0.996; *d* = 1.3 × 10^−3^	*rs* = 0.17, *p*_bonf_ = 1.000 *p* = 0.187; *d* = 0.34	*rs* = 0.18, *p*_bonf_ = 1.000 *p* = 0.151; *d* = 0.37	*rs* = 0.17, *p*_bonf_ = 1.000 *p* = 0.172; *d* = 0.34
Education (years)	–	*rs* = −0.10, *p*_bonf_ = 1.000 *p* = 0.435; *d* = −0.20	*rs* = −0.18, *p*_bonf_ = 1.000 *p* = 0.145; *d* = −0.37	*rs* = 0.13, *p*_bonf_ = 1.000 *p* = 0.301; *d* = 0.26	*rs* = −0.04, *p*_bonf_ = 1.000 *p* = 0.729; *d* = −0.08
ERQ reappraisal	–	–	*rs* = 0.32, *p*_bonf_ = 0.150 *p* = 0.010; *d* = 0.67	*rs* = −0.30, *p*_bonf_ = 0.255 *p* = 0.017; *d* = −0.63	*rs* = −0.23, *p*_bonf_ = 0.945 *p* = 0.063; *d* = −0.47
ERQ suppression	–	–	–	*rp* = 0.06, *p*_bonf_ = 1.000 *p* = 0.636; *d* = 0.12	*rp* = −0.03, *p*_bonf_ = 1.000 *p* = 0.789; *d* = −0.06
PSS-10	–	–	–	–	*rp* = 0.62, *p*_bonf_ = 7.815 × 10^-7^^*^ *p* = 5.210 × 10^-8^; *d* = 1.58

ERQ, Emotion Regulation Questionnaire; PSS-10, Perceived Stress Scale (10 items); OCI-R, Obsessive-Compulsive Inventory-Revised;

* *Statistically significant correlations*.

**Figure 1:**
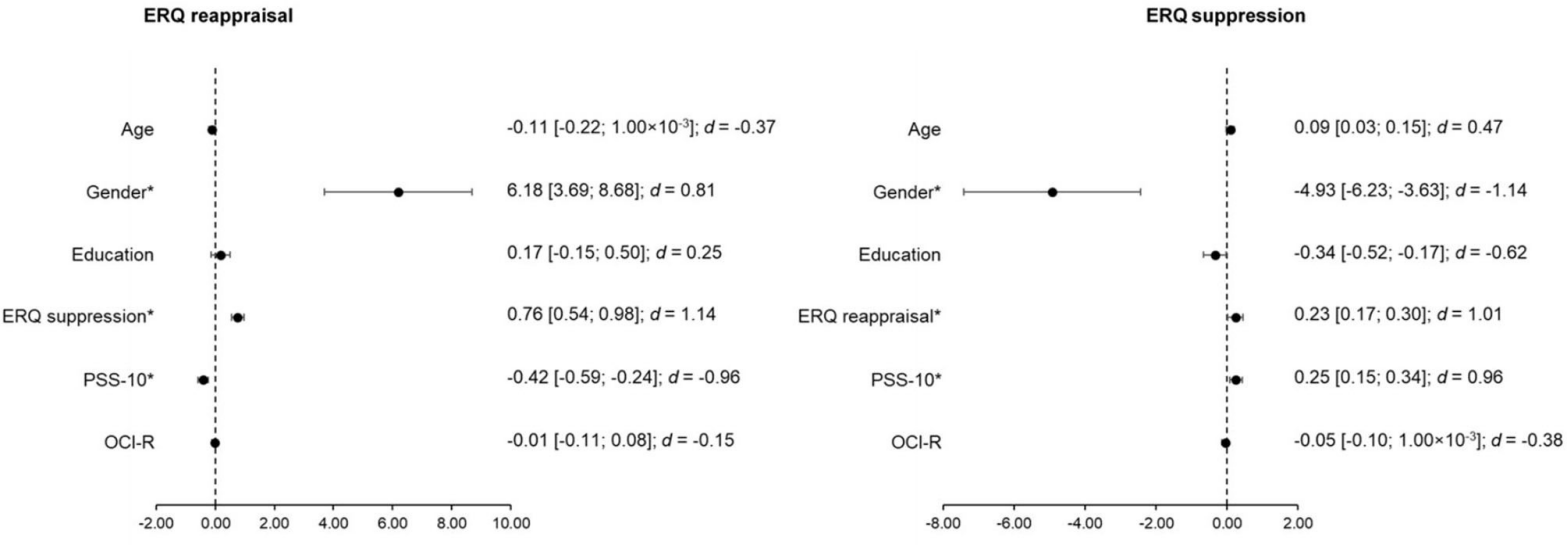
Representation of the estimates and standard error of the predictors for the multiple regression analyses for the ERQ reappraisal and ERQ suppression scores including the total sample. Gender is encoded as male−0 and female−1; ERQ, Emotion Regulation Questionnaire; PSS-10, Perceived Stress Scale (10 items); OCI-R, Obsessive-Compulsive Inventory-Revised; *d*—Cohen's effect size; *Statistically significant effects.

The regression model for the ERQ reappraisal score yielded statistical significance [*F*_(6, 56)_ = 3.53; *p* = 0.005; *R*^2^ = 0.27]. The ERQ reappraisal score was significantly predicted by gender (beta ± standard error = 6.18 ± 2.49; *t* = 2.48, *p* = 0.016, standardized beta = 0.33; effect size *d* = 0.81), the ERQ suppression score (0.76 ± 0.22; *t* = 3.47, *p* = 0.001, 0.44; *d* = 1.14), and the PSS-10 score (−0.42 ± 0.17; *t* = −2.40, *p* = 0.020,−0.38; *d* = −0.96). The regression model for the ERQ suppression score was also statistically significant [*F*_(6, 56)_ = 4.94; *p* = 4.000 × 10^−4^; *R*^2^ = 0.35]. The ERQ suppression score was significantly predicted by gender (−4.93 ± 1.30; *t* = −3.78, *p* = 4.000 × 10^−4^, −0.45; *d* = −1.14), the ERQ reappraisal score (0.23 ± 0.07; *t* = 3.47, *p* = 0.001, 0.40; *d* = 1.01), and the PSS-10 score (0.24 ± 0.10; *t* = 2.56, *p* = 0.013, 0.38; *d* = 0.96). Figure 1 represents the results of both regression models. In conclusion, increased values of ERQ reappraisal were associated with higher ERQ suppression scores. Female participants had higher values in ERQ reappraisal and lower values in ERQ suppression. Elevated values of PSS-10 corresponded to increased scores in ERQ suppression and decreased ERQ reappraisal scores.

**Figure 2:**
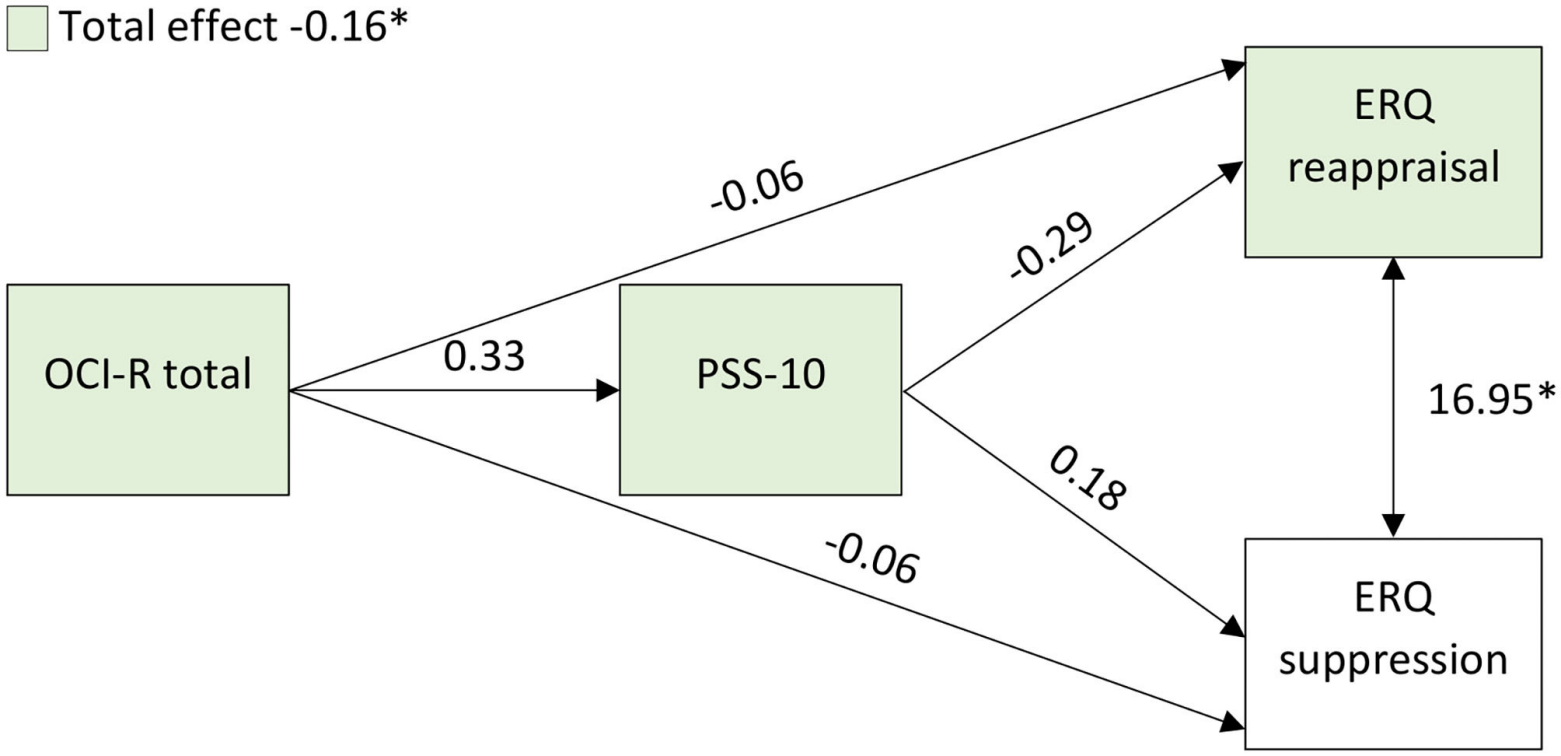
Representation of the mediation analysis results. The values represent the estimates. ERQ, Emotion Regulation Questionnaire; PSS-10, Perceived Stress Scale (10 items); OCI-R, Obsessive-Compulsive Inventory-Revised; *Statistically significant effects.

For the mediation analysis, the direct effect of OCI-R on ERQ reappraisal (beta ± standard error = −0.06 ± 0.09, *p* = 0.502; *d* = −0.17) and suppression (−0.06 ± 0.05, *p* = 0.217; *d* = −0.31) was not statistically significant. Moreover, the indirect effect of OCI-R on ERQ reappraisal (−0.09 ± 0.06, *p* = 0.116; *d* = −0.40) and suppression (0.06 ± 0.03, *p* = 0.075; *d* = 0.46) when mediated by PSS-10 was also not statistically significant. Nonetheless, the total effect (combination of direct and indirect effects) was statistically significant for the ERQ reappraisal (−0.16 ± 0.08, *p* = 0.036; *d* = −0.54) but not for the ERQ suppression score (-4.00 × 10^−3^ ± 0.04, *p* = 0.916; *d* = −0.03). Moreover, the ERQ reappraisal and suppression score had a statistically significant association (16.95 ± 5.55, *p* = 0.002; *d* = 0.83) (57). Figure 2 represents the mediation analysis results. In conclusion, the ERQ reappraisal score is explained by the direct effect of the OCI-R score combined with the OCI-R effect mediated by the PSS-10 score. Moreover, the ERQ reappraisal and suppression score influence each other.

## Discussion

In this study, we evaluated if stress and OC symptoms have a negative effect on emotion regulation measures in a sample composed of OCD and healthy participants. Our main results demonstrated that suppression and reappraisal abilities are predicted by gender and stress levels but not by OC symptoms. Moreover, we observed that the reappraisal score results from a combination of a direct effect of OC symptoms and an indirect effect of OC symptoms mediated by stress levels.

First, our results showed that OCD patients had reduced reappraisal scores in line with past findings (21–26). However, in contrast with these findings, we did not observe an augmented use of suppression in the OCD group. Most of the patients were under pharmacological treatment. Thus, they might have reduced the use of suppression to attenuate the emotional impact of obsessions and distress. However, the median Y-BOCS score indicates severe to extreme OC symptomatology despite the treatment. Moreover, some authors did not find increased suppression scores (21, 24, 26) even in OCD patients without medication. In this way, other factors may affect the suppression score in OCD individuals. On the other hand, the control participants included in this study may regularly use suppression strategies given the higher difficulty and cognitive cost in using reappraisal for emotion regulation (59–61). In agreement with this, we found that higher emotion reappraisal abilities were predicted by increased suppression scores and vice versa. Furthermore, there was a positive influence between reappraisal and suppression scores in the mediation analysis. Thus, our results might indicate that effective emotion regulation depends on the use of both strategies. Indeed, past findings showed that the frequent practice of reappraisal is not linked to reduced use of suppression strategies (62). Additionally, studies exploring the spontaneous use of emotion regulation strategies showed that reappraisal is not applied more often than suppression (63).

We also found augmented levels of perceived stress in the OCD group supporting the interplay between OCD and stress (1, 10, 14, 64). This outcome was further reinforced by a strong positive correlation between stress and OC scores in the total sample and the OCD group.

Both the suppression and reappraisal scores were predicted by gender and stress levels but not by the OC score. Moreover, the reappraisal score resulted from a combination of a direct effect of OC symptoms and an indirect effect of these symptoms mediated by perceived stress levels. Past researchers also reported that women express more their emotions and have more practice at successfully regulating them (29, 65), while men are culturally shaped to suppress some type of emotions (e.g., sadness and fear) (66). Thus, males might have more difficulties in identifying, accepting, and regulating emotions. Moreover, women use suppression strategies less frequently than men (67). Consistent with our findings, previous researchers also found that maladaptive strategies (suppression and rumination) and reappraisal were positively and negatively associated with stress symptoms, respectively (62, 68). Also, individuals under stressful conditions are more predisposed to the effects of negative emotional stimuli (69–71), and are ineffective in distracting themselves (69, 72) or reappraising their emotions (71, 73) when exposed to affective stimuli. Moreover, stress leads to the engagement of maladaptive strategies such as worry and rumination (33). Thus, individuals under stress may be more prone to use suppressing strategies. These findings may result from stress-induced impairment of cognitive processes (e.g., cognitive flexibility and inhibitory and goal-directed behavior) due to the disruption of prefrontal function (74). Thus, stress might inhibit the prefrontal cortical activity hampering the modulation of limbic regions (e.g., amygdala) during emotion regulation (18, 70). Indeed, these brain regions are also implicated in emotion regulation processes (32, 75). In summary, OCD individuals have elevated stress symptoms that might weaken their ability to use emotion reappraisal strategies. Their cognitive resources are impaired by stress leading to an increased response to negative emotions (59). Instead of reappraisal, they may choose more effortless maladaptive strategies (e.g., suppression and compulsions) (61) to regulate their emotions, leading to a rebound effect on distress and anxiety levels (27, 28, 31, 68, 76).

Our findings are limited by the lack of control for anxiety and depression levels. Both OC and stress symptoms are associated with anxious and depressed mood (77). Yap et al. (78) found that OCD severity was not associated with emotion regulation deficits when controlling for anxiety and depression scores. Moreover, Moore et al. (62) found associations between the ERQ scores and anxiety and depression symptoms. Thus, anxiety and depression might have a significant impact on emotional regulation (79, 80). Our results might have also been affected by the fact that most of the OCD patients selected for this study were medicated and some were frequenting psychotherapy sessions. Moreover, our study has a cross-sectional design hampering the analysis of stress and OC symptoms variations on emotional regulation. Future studies with cognitive-behavioral therapy for OCD and stress management might provide further insights. Additionally, our sample had a higher proportion of female individuals. However, the main conclusions were controlled for gender ratio. Finally, our results need to be replicated with larger samples to increase the study power. Indeed, some of our findings did not show a large effect size (differences between OCD and control groups in the reappraisal score, and the total effect of OC symptoms in the reappraisal score in the mediation analysis).

## Conclusion

This study provides a novel perspective of emotional regulation impairments in OCD. The reliance on suppression strategies and the difficulty in using reappraisal approaches are explained by stress levels and not directly explained by OC symptoms. Our conclusions support the inclusion of stress management in cognitive-behavioral therapy treatments to improve the processes of emotion regulation in OCD patients.

## Data Availability Statement

The raw data supporting the conclusions of this article will be made available by the authors, without undue reservation.

## Ethics Statement

The studies involving human participants were reviewed and approved by Comissão de Ética para a Saúde, Hospital de Braga and Subcomissão de Ética para as Ciências da Vida e da Saúde, University of Minho. The patients/participants provided their written informed consent to participate in this study.

## Author Contributions

SF, BC, MS, RV, and MP-P: study design, data collection, data analysis, and manuscript writing. NS, MP-P, and PM: study design, manuscript writing, and study supervision. All authors contributed to the article and approved the submitted version.

## Conflict of Interest

The authors declare that the research was conducted in the absence of any commercial or financial relationships that could be construed as a potential conflict of interest.

## Abbreviations

OCD: obsessive-compulsive disorder
HPA: hypothalamic-pituitary-adrenal
OC: obsessive-compulsive
Y-BOCS: Yale-Brown Obsessive Compulsive Scale
ERQ: Emotion Regulation Questionnaire
OCI-R: Obsessive-Compulsive Inventory-Revised
PSS-10: 10-items Perceived Stress Scale.
