# Supplementary material for: Stress Influences the Effect of Obsessive-Compulsive Symptoms on Emotion Regulation
Source: Front Psychiatry. 2021 Jan 20;11:594541. doi: 10.3389/fpsyt.2020.594541 (PMC7854917; doi:10.3389/fpsyt.2020.594541)
Supplement: Supplementary file 1 [file Table_1.docx]

| **Table 1** Results of Pearson’s (*rp*; normally distributed variables) and Spearman’s (*rs*; variables not normally distributed) among demographic and psychometric variables for the obsessive-compulsive group (*p*_bonf_ - *p*-value after Bonferroni correction; *d* – Cohen’s effect size). | | | | | |
| --- | --- | --- | --- | --- | --- |
|  | Education (years) | ERQ reappraisal | ERQ suppression | PSS-10 | OCI-R total |
| Age  (years) | *rs* = -0.16, *p*_bonf_ = 1.000  *p* = 0.317; *d* = -0.32 | *rs* = -0.03, *p*_bonf_ = 1.000  *p* = 0.855; *d* = -0.06 | *rs* = 0.21, *p*_bonf_ = 1.000  *p* = 0.174; *d* = 0.43 | *rs* = -4.00×10^-3^, *p*_bonf_ = 1.000  *p* = 0.980; *d* = -0.01 | *rs* = 0.09, *p*_bonf_ = 1.000  *p* = 0.583; *d* = 0.18 |
| Education (years) | - | *rs* = -0.12, *p*_bonf_ = 1.000  *p* = 0.444; *d* = -0.24 | *rs* = -0.19, *p*_bonf_ = 1.000  *p* = 0.224; *d* = -0.39 | *rs* = 0.13, *p*_bonf_ = 1.000  *p* = 0.406; *d* = 0.26 | *rs* = -0.10, *p*_bonf_ = 1.000  *p* = 0.509; *d* = -0.20 |
| ERQ reappraisal | - | - | *rp* = 0.18, *p*_bonf_ = 1.000  *p* = 0.257; *d* = 0.37 | *rp* = -0.39, *p*_bonf_ = 0.150  *p* = 0.010; *d* = -0.85 | *rp* = -0.23, *p*_bonf_ = 1.000  *p* = 0.136; *d* = -0.47 |
| ERQ suppression | - | - | - | *rp* = 0.03, *p*_bonf_ = 1.000  *p* = 0.854; *d* = 0.06 | *rp* = -0.01, *p*_bonf_ = 1.000  *p* = 0.935; *d* = -0.02 |
| PSS-10 | - | - | - | - | ***rp* = 0.55, *p*_bonf_ = 0.002***  ***p* = 1.693×10^-4^; *d* = 1.32** |
| ERQ – Emotion Regulation Questionnaire; PSS-10 – Perceived Stress Scale (10 items); OCI-R – Obsessive-Compulsive Inventory-Revised; *Statistically significant correlations. | | | | | |
