# Supplementary material for: Stress Influences the Effect of Obsessive-Compulsive Symptoms on Emotion Regulation
Source: Front Psychiatry. 2021 Jan 20;11:594541. doi: 10.3389/fpsyt.2020.594541 (PMC7854917; doi:10.3389/fpsyt.2020.594541)
Supplement: Supplementary file 2 [file Table_2.docx]

| **Table 2** Results of Pearson’s (*rp*; normally distributed variables) and Spearman’s (*rs*; variables not normally distributed) among demographic and psychometric variables for the control group (*p*_bonf_ - *p*-value after Bonferroni correction; *d* – Cohen’s effect size). | | | | | |
| --- | --- | --- | --- | --- | --- |
|  | Education (years) | ERQ reappraisal | ERQ suppression | PSS-10 | OCI-R total |
| Age (years) | *rs* = -0.11, *p*_bonf_ = 1.000  *p* = 0.632; *d* = -0.22 | *rs* = 0.14, *p*_bonf_ = 1.000  *p* = 0.520; *d* = 0.28 | *rs* = 0.12, *p*_bonf_ = 1.000  *p* = 0.605; *d* = 0.24 | *rs* = 0.32, *p*_bonf_ = 1.000  *p* = 0.148; *d* = 0.67 | *rs* = 0.22, *p*_bonf_ = 1.000  *p* = 0.328; *d* = 0.45 |
| Education (years) | - | *rp* = -0.04, *p*_bonf_ = 1.000  *p* = 0.871; *d* = -0.08 | *rp* = -0.11, *p*_bonf_ = 1.000  *p* = 0.619; *d* = -0.22 | *rp* = 0.07, *p*_bonf_ = 1.000  *p* = 0.743; *d* = 0.14 | *rp* = -0.04, *p*_bonf_ = 1.000  *p* = 0.858; *d* = -0.08 |
| ERQ reappraisal | - | - | *rp* = 0.54, *p*_bonf_ = 0.150  *p* = 0.010; *d* = 1.28 | *rp* = 0.24, *p*_bonf_ = 1.000  *p* = 0.272; *d* = 0.49 | *rp* = 0.18, *p*_bonf_ = 1.000  *p* = 0.426; *d* = 0.37 |
| ERQ suppression | - | - | - | *rp* = 0.22, *p*_bonf_ = 1.000  *p* = 0.320; *d* = 0.45 | *rp* = 0.02, *p*_bonf_ = 1.000  *p* = 0.919; *d* = 0.04 |
| PSS-10 | - | - | - | - | *rp* = 0.48, *p*_bonf_ = 0.420  *p* = 0.028; *d* = 1.09 |
| ERQ – Emotion Regulation Questionnaire; PSS-10 – Perceived Stress Scale (10 items); OCI-R – Obsessive-Compulsive Inventory-Revised. | | | | | |
